# [Expression of Concern] Gene therapy for human colorectal cancer cell lines with recombinant adenovirus 5 based on loss of the insulin-like growth factor 2 imprinting

**DOI:** 10.3892/ijo.2025.5820

**Published:** 2025-11-18

**Authors:** Huiling Sun, Yuqin Pan, Bangshun He, Qiwen Deng, Rui Li, Yeqiong Xu, Jie Chen, Tianyi Gao, Houqun Ying, Feng Wang, Xian Liu, Shukui Wang

Int J Oncol 46: 1759-1767, 2015; DOI: 10.3892/ijo.2015.2852

Following the publication of the above paper, it was drawn to the Editor's attention by a concerned reader that, for the immunohistochemical data shown in Fig. 2B and C, the PBS/TUNEL panel in Fig. 2B appeared to be strikingly similar to the PBS/E1A panel shown in Fig. 2C. Furthermore, for the E1A experiments portrayed in Fig. 2C, portions of the data panels shown for the H101 and E1A groups also appeared to be strikingly similar, albeit with rotation of one of the panels.

The authors were contacted by the Editorial Office to offer an explanation for this possible anomaly in the presentation of the data in this paper, although up to this time, no response from them has been forthcoming. Owing to the fact that the Editorial Office has been made aware of potential issues surrounding the scientific integrity of this paper, we are issuing an Expression of Concern to notify readers of this potential problem while the Editorial Office continues to investigate this matter further.

